# Biosynthetic pathway and optimal conditions for the production of indole-3-acetic acid by an endophytic fungus, *Colletotrichum fructicola* CMU-A109

**DOI:** 10.1371/journal.pone.0205070

**Published:** 2018-10-18

**Authors:** Tosapon Numponsak, Jaturong Kumla, Nakarin Suwannarach, Kenji Matsui, Saisamorn Lumyong

**Affiliations:** 1 Department of Biology, Faculty of Science, Chiang Mai University, Chiang Mai, Thailand; 2 Graduate School of Sciences and Technology for Innovation, Yamaguchi University, Yamaguchi, Japan; 3 The Center of Excellence for Renewable Energy, Chiang Mai University, Chiang Mia, Thailand; Universita degli Studi di Pisa, ITALY

## Abstract

Endophytic fungi are known to produce indole-3-acetic acid (IAA), which can stimulate plant growth. Twenty-seven isolates of endophytic fungi were isolated from *Coffea arabica* in northern Thailand. Only one isolate (CMU-A109) produced IAA *in vitro*. This isolate was identified as *Colletotrichum fructicola* based on morphological characteristics and molecular phylogenetic analysis of a combined five loci (internal transcribed spacer of ribosomal DNA, actin, β-tubulin 2, chitin synthase and glyceraldehyde-3-phosphate dehydrogenase genes). Identification of a fungal IAA production obtained from indole 3-acetamide (IAM) and tryptophan 2-monooxygenase activity is suggestive of IAM routed IAA biosynthesis. The highest IAA yield (1205.58±151.89 μg/mL) was obtained after 26 days of cultivation in liquid medium supplemented with 8 mg/mL L-tryptophan at 30°C. Moreover, the crude fungal IAA could stimulate coleoptile elongation of maize, rice and rye. This is the first report of IAA production by *C*. *fructicola* and its ability to produce IAA was highest when compared with previous reports on IAA produced by fungi.

## Introduction

Endophytic fungi are defined as the fungi that colonize plant tissues without causing any disease-related symptoms [1–3]. Colonization by endophytic fungi can improve the ecological adaptation of the host plant by enhancing its growth and tolerance to biotic and abiotic stresses [4, 5]. Several studies have reported the potential of endophytic fungi to produce bioactive compounds that protect the host plant against microbial influences and promote plant growth in both volatile and non-volatile substance productions [6–8]. Many studies have shown that plant growth promotion may be attributed to the secretion of plant-growth promoting secondary metabolites (phytohormones e.g. auxins, cytokinins, ethylene and gibberellins, and siderophore) and the ability to mobilize insoluble phosphate and provide nitrogen to their host plants by endophytic fungi [9–13].

Indole-3-acetic acid (IAA) is a dominant type of auxin found in plants and is involved in the growth responses of plants for the regulation of cell elongation, cell division, cell differentiation and root initiation [14, 15]. IAA has been produced not only in plants, but also by plant-associated microorganisms including ectomycorrhizal, endophytic, pathogenic, phyllosphere and rhizospheric microbes [16–20]. The main precursor for the IAA synthesis is L-tryptophan (L-Trp) and there are at least five different pathways that have been described for the IAA biosynthetic pathway in microorganisms including the indole-3-acetamide (IAM), indole-3-pyruvate (IPyA), tryptamine (TAM), indole-3-acetonitrile (IAN), and tryptophan side-chain oxidase (TSO) pathways [21, 22]. The IAM, IAN and TPyA pathways are considered the major IAA biosynthesis pathways in bacteria [23]. IAA production via TSO pathways was only reported in *Pseudomonas fluorescens* strain CHA0 [24]. Some fungi (e.g. *C*. *acutatum*, *Fusarium proliferatum*, *F*. *fujikuroi*, *F*. *oxysporum*, *Muscodor cinnamomi*, *Piriformospora indica*, *Ustilago esculenta* and *U*. *maydis*) and yeasts (*Aureobasidium pullulans*, *Cryptococcus flavus*, *Hannaella coprosmaensis*, *Pseudozyma aphidis*, *Rhodosporidium paludigenum*, *Rhodotorula graminis*, *Saccharomromyces cerevisiae* and *Sporisorium reilianum*) mainly use IAM and IPyA pathways for IAA biosynthesis [25–33]. However, there is still a need for further research on the IAA produced by endophytic fungi, as well as investigations of their IAA biosynthesis pathway. In this study, the endophytic fungi were isolated from coffee plants and their *in vitro* IAA production ability was investigated. The IAA synthesis pathway of the IAA producer fungi was identified. The highest IAA producer was selected and the optimal conditions for IAA production were determined. Moreover, the biological effect of crude fungal IAA was also evaluated.

## Materials and methods

### Ethics statement

No specific permits were required to carry out research in the coffee plantation. This area did not involve endangered or protected species in Thailand. Permission to coffee plant samples was granted by the Highland Research and Training Center, Faculty of Agriculture, Chiang Mai University, Thailand.

### Fungal isolation

Endophytic fungi were isolated from healthy mature leaves and stems of coffee plants (*Coffea arabica* L.) collected from Khun Changkean of Highland Research and Training Center, Faculty of Agricultural, Chiang Mai University, Thailand (18°50'43.0"N, 98°54'26.0"E) in November 2012. The coffee leaves and stems were washed in running tap water for 15 min and cut into small pieces (5 mm^2^ leaf and 1 mm length stem). The cut samples were triple surface-sterilized method (70% ethanol for 1 min, 2% sodium hypochlorite for 3 min and 95% ethanol for 30 sec) [34] under a laminar flow hood. Twenty-five leaf and stem pieces were placed on potato dextrose agar (PDA) supplemented with 50 ppm of chloramphenicol and 35 ppm of Rose Bengal. The plates were incubated at 25°C. The fungi growing out from the tissue samples were transferred to fresh PDA plates. The pure cultures were preserved on PDA slants for the short-term and 20% glycerol at -20°C for the long-term at The Culture Collection of the Sustainable Development of Biological Resources (SDBR) Laboratory, Faculty of Science, Chiang Mai University, Chiang Mai and Thailand Bioresource Research Center, Pathum Thani Province, Thailand.

### Identification of IAA producing fungal endophyte

#### Morphological studies

Conventional morphological characters were used to tentatively identify the selected IAA producing endophytic fungi. Colony characteristics including aerial mycelium, density and pigment production were recorded. Micromorphological characteristics were examined using a light microscope (Olympus CX51, Japan). Size data of the anatomical features are based on at least 50 measurements of each structure.

#### Molecular studies

Molecular techniques were used to confirm the identification of IAA producing endophytic fungi. Genomic DNA was extracted from one week-old fungal mycelia on PDA (1–5 mg) using a DNA Extraction Mini Kit (FAVORGEN, Taiwan) following the manufacturer’s protocol. The internal transcribed spacer (ITS) region of ribosomal DNA (rDNA), actin (ACT), β-tubulin 2 (TUB2), chitin synthase (CHS-1) and glyceraldehyde-3-phosphate dehydrogenase (GAPDH) genes were amplified with primers and annealing temperatures following the previous studies [35−38] as presented in Table 1. Polymerase chain reaction (PCR) was performed in 20 μL reaction containing 1.0 μL DNA template, 1.0 μL of each forward and reverse primers, 10.0 μL 2X Quick Taq HS DyeMix (TOYOBO, Japan) and 7 μL deionized water, and the following thermal conditions: 94°C for 2 min, followed by 35 cycles of 94°C for 2 min, the temperatures dependent on the amplified gene (Table 1) for 1 min and 72°C for 1 min, and a final 72°C for 10 min on a peqSTAR thermal cycler (PEQLAB Ltd., UK). PCR products were checked on 1% agarose gels stained with ethidium bromide under UV light. PCR products were purified using a PCR clean up Gel Extraction NucleoSpin Gel and PCR Clean-up Kit (Macherey-Nagel, Germany). The purified PCR products were directly sequenced. Sanger sequencing was carried out by 1^ST^ Base Company (Kembangan, Malaysia) using the PCR primers mentioned above. Sequences were used to query GenBank via BLAST (http://blast.ddbj.nig.ac.jp/top-e.html).

**Table 1:** Details of primers, annealing temperature and the obtained product size of the amplification of gene targets in this study.

Region	Primer name	Orientation	Reference	Annealing temperature (°C)	Obtained product size (bp)
ITS	ITS5	Forward	[35]	54	589
	ITS4	Reverse			
ACT	ACT512F	Forward	[36]	58	285
	ACT783R	Reverse			
TUB2	T1	Forward	[37]	52	743
	T22	Reverse			
GAPDH	GSF1	Forward	[38]	60	278
	GSR1	Reverse			
CHS-1	CHS-79F	Forward	[36]	50	297
	CHS-345R	Reverse			

For phylogenetic analysis, the sequences obtained from this study and from previous studies along with sequences from GenBank database were used. The multiple sequence alignment was carried out using MUSCLE [39]. A maximum likelihood (ML) phylogenetic tree from a combined ITS, ACT, TUB2, GAPDH and CHS-1 sequences was constructed using RAxML v7.0.3 [40], applying the rapid bootstrapping algorithm for 1000 replications using the GTRGAMMA model. The ML trees were viewed with TreeView32 [41]. Bayesian phylogenetic analyses were carried out using the Metropolis-coupled Markov chain Monte Carlo (MCMCMC) method in MrBayes version 3.2 [42].

### Determination of fungal indole compound production

One fungal disc (5 mm in diameter) was inoculated into 5 mL of potato dextrose broth (PDB) supplemented with 2 mg/mL L-Trp (Sigma-Aldrich, Germany), pH 6.0, in 18 (internal diameter) × 180 mm test tubes and incubated in the dark on a shaker for one week at room temperature (25±2ºC). The fungal culture was collected by centrifugation at 1250 × g for 5 min. The colorimetric assay [43] was performed to determine fungal indole compound production. The fungal culture was mixed with Salkowski’s reagent (1 mL of 0.5 mol/L FeCl_3_ in 50 mL of 35% HClO_4_) in ratios of 1:2 (v/v), respectively, and incubated in the dark for 30 min. The pink to red color was considered positive for IAA production. Three replications were made.

### Extraction of fungal indole metabolites

The fungal IAA supernatant was acidified to pH 4.0 by using 1 mol/L HCl and mixed twice with ethyl acetate at double the volume of the supernatant. The extracted ethyl acetate was dried using a rotary evaporator. The crude extract was dissolved in methanol and kept at -20°C.

### Detection and quantification of fungal IAA and related indole compounds

#### Thin layer chromatography

The crude ethyl acetate extract of fungal IAA was applied to of thin layer chromatography (TLC) plate (Silica gel G F257, thickness 0.25 mm, Merck, Germany). The chromatogram was performed in the mobile phase that contained *n*-hexane: ethyl acetate: isopropanol: acetic acid (40:20:5:1, v/v/v/v) following the study of Chung et al. [16]. Spots with R_f　_values identical to standards of L-Trp, IAA (Sigma-Aldrich, Germany), tryptophol (TOL; Wako Pure Chemicals, Japan), IPyA (Sigma-Aldrich, Germany), IAM (Wako Pure Chemicals, Japan), TAM (Sigma-Aldrich, Germany), indole-3-lactic acid (ILA; Wako Pure Chemicals, Japan) and IAN (Sigma-Aldrich, Germany) were identified under UV light (254 nm) and after sprayed with Ehmann’s [44], Ehrlich’s [44] and Salkowski’s reagents.

#### High performance liquid chromatography

The quantification of fungal IAA was performed with high performance liquid chromatography (HPLC) according to the method described by Szkop and Bielawski [45] with some modifications. The Shimadzu Prominence UFLC system was used, equipped with an LC-20 AD pump, SIL20ACHT autosampler, CTO-20 AC column oven, CBM-20A system controller and SPD-20A photodiode array detector. Ten microliters of the sample were injected to the Mightysil RP-18 (250 × 4.6 mm, 5 μm) column heated at 40°C. The gradient elution was applied. The solvent A consisted of 2.5 : 97.5% (v/v) acetic acid : deionzed water, pH 3.8 (the final pH was adjusted by 10 mol/L KOH) and solvent B consisted of 80 : 20% (v/v) acetonitrile : deionzed water. The mobile phase start with solvent A: solvent B at 80 : 20%, changing to 50 : 50%, 0 : 100% and 80 : 20% at 25, 31 and 33 min, respectively. The total run time was 40 min and a flow rate of mobile phase was set to 0.5 mL/min. The injection volume was 10 μL. The detection wavelengths were 280 and 350 nm. Retention times of the samples were determined by comparing them with the L-Trp, IAA, TOL, IPyA, IAM, TAM, ILA and IAN standards and further by co-injections. Quantification of fungal IAA and related indole compounds were carried out with the calibration curve constructed with various indole compounds.

### Determination of IAA biosynthesis pathway of selected IAA producing isolate

#### IAA production from intermediate indole compound cultivation

To analyze the IAA biosynthetic pathway of the selected fungus, five mycelial discs were cultivated with 5 mL of basal liquid medium (2.0 g NaNO_3_, 0.5 g MgSO_4_·7H_2_O, 0.5 g KCl, 0.01 g FeSO_4_·7H_2_O, 10.0 g glucose and 15.0 g agar per liter of distilled water, pH 6.0) supplemented with 2 mg/mL of each indole intermediate compound (IAM, IAN, IPyA and TAM) in 18 × 180 mm test tubes at 30°C under shaking conditions at 120 rpm in the darkness for one week. Cultured filtrates were sampled every day and were then extracted. The crude extracts were examined in terms of the IAA and intermediate indole compound production by HPLC techniques, as has been described above. Three replications were made.

#### Tryptophan 2-monooxygenase activity

The fungus was cultivated in the basal liquid medium supplemented with 2 mg/mL of L-Trp at 30°C under shaking conditions at 120 rpm in the darkness for five days. Mycelia were filtrated and washed twice with Tris-HCl buffer (50 mmol/L, pH 7.8) and resuspended in 4 mL of the same buffer. Mycelia were lysed by sonication with an Ultrasonic disruptor UD-201 (Tomy Seiko, Japan). After sonication, lysate was centrifuged (1250 × g, 10 min, 4°C) and clear supernatant was used as a source of enzyme. Determination of tryptophan 2-monooxygenase activity followed the method described by Mujahid et al. [46] with some modifications. Enzyme assay was carried out in the final volume of 1 mL Tris buffer (50 mmol/L, pH 7.8) containing 0.5 mL of L-Trp and 0.5 mL of enzyme extract. Reaction mixture was incubated at 30°C for 25 min and then stopped by addition of 100 μL of 5 mol/L HCl. Reaction mixture was centrifuged and the supernatant was collected. The formation of IAM was analyzed by HPLC techniques as mentioned above. Three replications were made.

### Optimal conditions for fungal IAA production

One fungal isolate (CMU-A109) showing the ability to produce IAA was obtained and used to identify the optimal conditions for IAA production, including L-Trp concentration, temperature and cultivation period. The effect of L-Trp concentration on IAA produced by isolate CMU-A109 was studied by inoculating fungal disc (5 mm in diameter) into 18 × 180 mm test tubes containing 5 mL PDB supplemented with different concentrations of L-Trp (0, 2, 4, 6, 8 and 10 mg/mL) and shaken in the darkness at room temperature (25±2ºC) for one week. The fungal supernatants were then harvested and the estimated levels of IAA production were measured by HPLC. Three replications were made. The concentration of L-Trp that gave the highest level of IAA was selected for further experiments.

Different temperatures for fungal IAA production were examined. Cultivation was performed in the darkness at various temperatures (22ºC, room temperature (25±2ºC), 30ºC and 37°C) on a shaker at 120 rpm. The fungal supernatants were harvested and the amount of IAA was determined after one week of cultivation. This treatment was carried out in triplicate.

The effects of the cultivation period on IAA production were studied. The fungal discs were cultivated in liquid medium and incubated in the dark on a shaker with 120 rpm at 30°C for 30 days. The amount of fungal IAA was evaluated every 2 days. Three replications were made.

### Biological activities of fungal IAA

Biological activity of fungal IAA produced by isolate CMU-A109 was determined with the coleoptile elongation of corn (*Zea mays* L.), rice (*Oryza sativa* L.) and rye (*Secale cereal* L.) [17, 20]. Crude fungal IAA was dissolved in 0.5 mL of 0.1 mol/L NaOH, and then diluted with sterile distilled water. Crude fungal IAA solution (containing 20 and 30 μg/mL of IAA and IAM, respectively) was used in this experiment. The positive control consisted of the stock solution of 20 μg/mL IAA, 30 μg/mL_,_ IAM and IAA mixed with IAM (20 μg/mL IAA mixed with 30 μg/mL IAM), and sterile distilled water was used as the negative control. The seed were surface disinfested in a mixture of 0.2% Tween 80 and 2% sodium hypochlorite for 3 min, followed by rinsing three times in sterile water. The surface disinfested seeds were placed on 1.5% water agar and incubated at 25ºC. After 3 days, 1.5−2.0 mm tips of the coleoptiles from seedlings non-colonized by fungi and bacteria were removed and adjusted to 10 mm lengths. Sections of coleoptiles were floated in distilled water for 2 h before being used. For each treatment, ten coleoptile segments were floated in a 9 cm Petri dish containing 20 mL of each solution and were then incubated in the dark at 25ºC. After 24 h, the length of the coleoptile segments was measured. Each treatment was conducted with five replicates.

### Statistical analysis

Statistical analyses were carried out by one-way analysis of variance (ANOVA) using SPSS program version 20 for Windows. Tukey’s range test was used to determine significant differences (*P*<0.05) between the mean values of each treatment.

## Results

### Fungal isolation and identification of IAA producing isolate

Twenty-seven endophytic fungi isolates were obtained from arabica coffee. Ten isolates were obtained from stem tissues and 17 isolates were obtained from leaf tissues. Only one isolate (CMU-A109) displayed positive IAA production when tested by colorimetric assay of Salkowski’s reagent as indicated by red color formation, while the uncultivated medium displayed a negative reaction.

**Fig 1:**
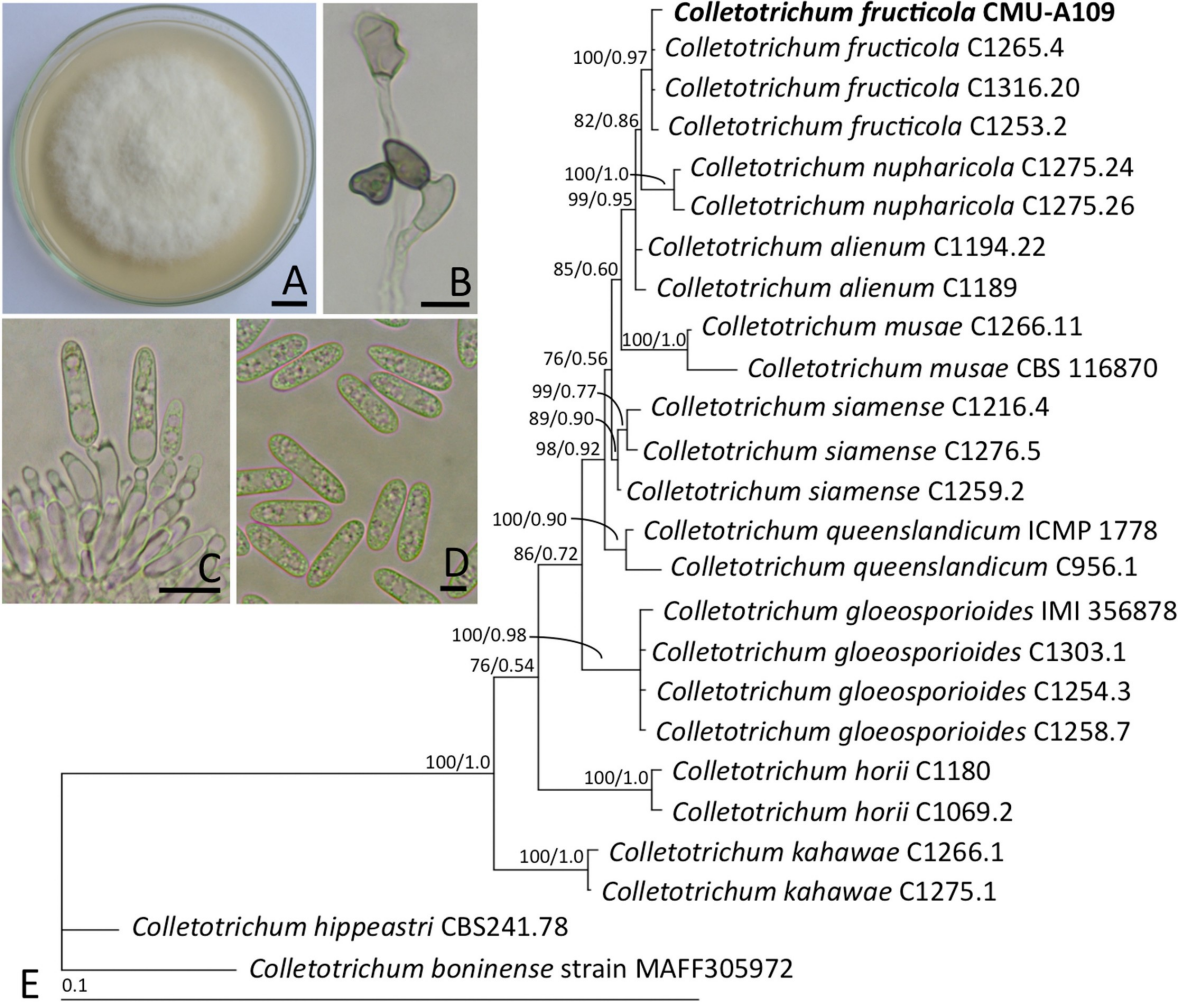
Morphological characteristics and phylogenetic tree of *Colletotrichum fructicola* CMU-A109. A. Colony on potato dextrose agar at 25°C for one week. B. Appressoria. C. Conidiophore and conidia. D. Conidia. E. Phylogram derived from maximum likelihood analysis of a combined five-gene loci (ITS, ACT, TUB2, CHS-1 and GAPDH) from *Colletotrichum fructicola* CMU-A109 and related species. *Colletotrichum boninense* and *Colletotrichum hippeastri* were used as the outgroup. The numbers above branches represent maximum likelihood bootstrap percentages (left), Bayesian posterior probabilities (right) and the species described in this study is shown in bold. Bar A = 10 mm, B and C = 5 μm, D = 1 μm, E = the number of nucleotide substitutions per site.

Colonies of isolate CMU-A109 on PDA grew to 80−85 mm at 25°C in the darkness after one week (Fig 1A). Colonies were cottony, white to pale grey and the reverse side was white to pale yellow in color. Sclerotia were absent. Mycelia were superficial and immersed. Hyphae were branched, septate, and hyaline to dark brown in color and 2−3 μm wide. Appressoria were 4.0−9.5 × 3.5−7.5 μm, mostly formed from mycelia, they were brown to dark brown in color, ovoid, clavate and slightly irregular to irregular in shape while often becoming complex with age (Fig 1B). Conidiophores were reduced to conidiogenous cells. Conidiogenous cells were cylindrical to ampulliform, hyaline, smooth, straight to curved and wider at the base, 7−18 × 1−3 μm (Fig 1C). Conidia were 9.7−14 × 3−4.5 μm (n = 50), commonly one-celled, smooth-walled with a large guttule at the centre, hyaline, cylindrical with obtuse to slightly rounded ends that were sometimes oblong (Fig 1D). Based on morphological observations, this fungal isolate was initially identified as belonging to the *Colletotrichum gloeosporioides* species complex [47, 48].

Molecular methods were used to confirm the identity of the isolate. The ITS, ACT, TUB2, CHS-1 and GAPDH sequences of isolate CMU-A109 were deposited in GenBank database under accession KT378065, MG717492, MG717493, MG717494 and MG717495, respectively. A phylogram of the combined five-gene loci (ITS, ACT, TUB2, CHS-1 and GAPDH) is presented in Fig 1E. Our phylogenetic results assigned isolate CMU-A109 to *C*. *fructicola* species. The species was clearly distinguished from other *Colletotrichum* species and formed a sister taxon to *C*. *nupharicola* with BS support of 82% and PP value support of 0.86. Therefore, isolate CMU-A109 was identified as *C*. *fructicola* based on molecular characteristics. A pure culture was deposited in the Culture Collection of the SDBR Laboratory, Faculty of Science, Chiang Mai University and Thailand Bioresource Research Center, Thailand under number SDBR-CMU-A109 and TBRC9265, respectively.

### Detection and quantification of IAA production by *Colletotrichum fructicola* CMU-A109

**Fig 2:**
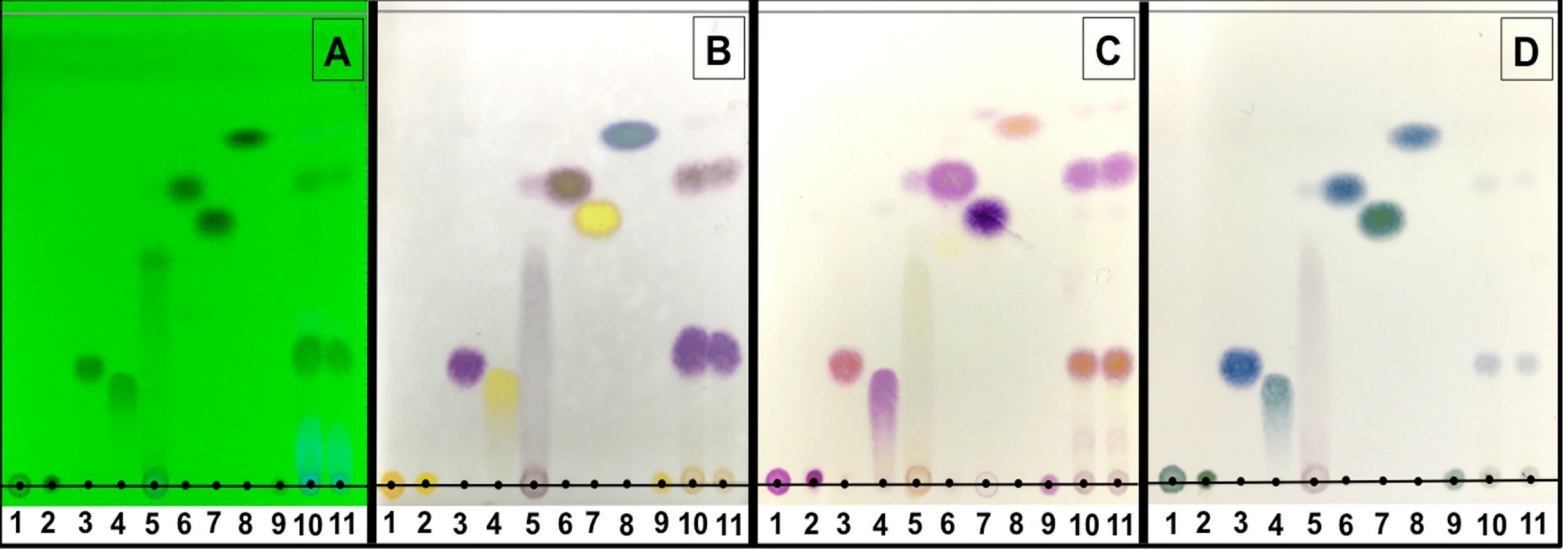
Identification of indole compounds produced by *Colletotrichum fructicola* CMU-A109 using thin layer chromatography technique under UV light (A), and chromogenic reaction reagents; Salkowski (B), Ehrlich (C) and Ehmann (D). Lane 1 = L-Trp, Lane 2 = TAM, Lane 3 = IAM, Lane 4 = ILA, Lane 5 = IPyA, Lane 6 = IAA, Lane 7 = TOL, Lane 8 = IAN, Lane 9 = uncultivated liquid medium, Lanes 10 and 11 = crude extract of fungal culture.

A combination of TLC and chromogenic reagents can provide another means for the verification of indole derivatives. The R_f_ values of each indole compound, crude fungal culture and crude uncultivated medium, which were applied in UV and chemical reagents, are presented in Fig 2 and Table 2. L-Trp and TAM were not separated under the conditions used (R_f_ = 0.0). IPyA was easily resolved into bands corresponding to IAA and other unknown compounds that are indicative of the instability of IPyA. The TLC chromatogram of a crude extract obtained from *C*. *fructicola* CMU-A109 revealed the same R_f_ values with those of IAM (0.24) and IAA (0.68) standards under UV light and spraying by Salkowski’s, Ehrlich’s and Ehmann’s reagents.

**Table 2:** Identification of indole compounds extracted from a culture of *Colletotrichum fructicola* CMU-A109 using chromogenic reagents after TLC separation.

Sample	Lane	Rf value	UV	Chromogenic reagent		
				Salkowski	Ehrlich	Ehmann
L-Trp	1	0.00	+	Yellow	Violet	Blue green
TAM	2	0.00	+	Yellow	Violet	Green
IAM	3	0.24	+	Violet	Violet pink	Blue
ILA	4	0.21	+	Yellow	Violet	Blue
IPyA	5	0.48	+	−	−	−
IAA	6	0.68	+	Violet red	Violet red	Blue
TOL	7	0.58	+	Yellow	Violet blue	Blue green
IAN	8	0.78	+	Blue green	Orange pink	Blue
Crude uncultured medium	9	0.00	+	Yellow	Violet	Blue green
Crude fungal culture	10	0.24	+	Violet	Violet pink	Blue
		0.68	+	Violet red	Violet pink	Blue
Crude fungal culture	11	0.24	+	Violet	Violet pink	Blue
		0.68	+	Violet red	Violet pink	Blue

“−” = no reaction.

**Fig 3:**
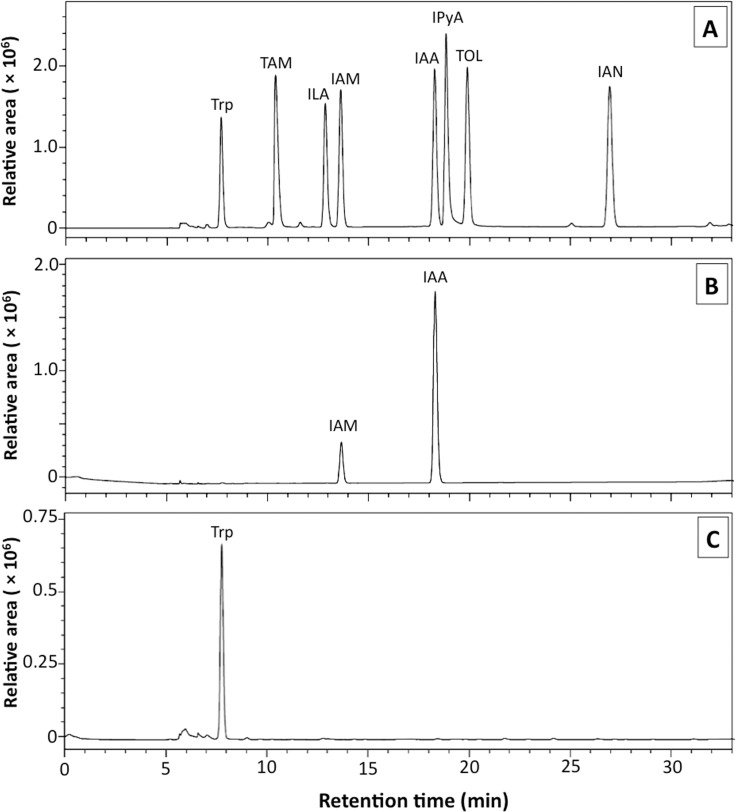
Identification of indole compounds produced by *Colletotrichum fructicola* CMU-A109 using high performance liquid chromatography technique. A. Indole compounds standard, B. Crude extract of fungal culture, C. Uncultivated liquid medium.

The HPLC analysis was conducted to more precisely identify the fungal IAA and other indole compounds. Under the relevant conditions, the retention times of L-Trp, TAM, ILA, IAM, IAA, IPyA, TOL, and IAN were 7.7, 10.4, 12.9, 13.7, 18.4, 19.1, 20.1 and 27.2 min, respectively (Fig 3A). The ethyl acetate extract of *C*. *fructicola* CMU-A109 showed a peak that corresponded to the IAA standard, with a maximum absorption value of 279 nm (Fig 3B). The identification of fungal IAA was confirmed by a co-injection with the IAA standard. The fungal IAA levels were also quantified by HPLC. *Colletotrichum fructicola* isolate CMU-A109 produced IAA at the level of 662.96±56.18 μg/mL. The HPLC chromatogram presented fungal IAM in crude fungal culture with a maximum absorption value of IAM at 230 nm. Identification of fungal IAM was confirmed by co-injection with the IAM standard. No correspondence of the IAA and IAM peaks was presented in the uncultivated medium extract (Fig 3C).

### Determination of IAA biosynthesis pathway of *Colletotrichum fructicola* CMU-A109

#### IAA production from intermediate indole compound

The IAA production by *C*. *fructicola* CMU-A109 by IAM, TAM, IPyA and IAN was examined by HPLC techniques. Fungal IAA was presented in the crude fungal culture with IAM (S1 Fig). No correspondence of the IAA peak was presented in the crude fungal culture with TAM, IPyA and IAN. Therefore, *C*. *fructicola* CMU-A109 produced IAA from IAM.

#### Tryptophan 2-monooxygenase activity

**Fig 4:**
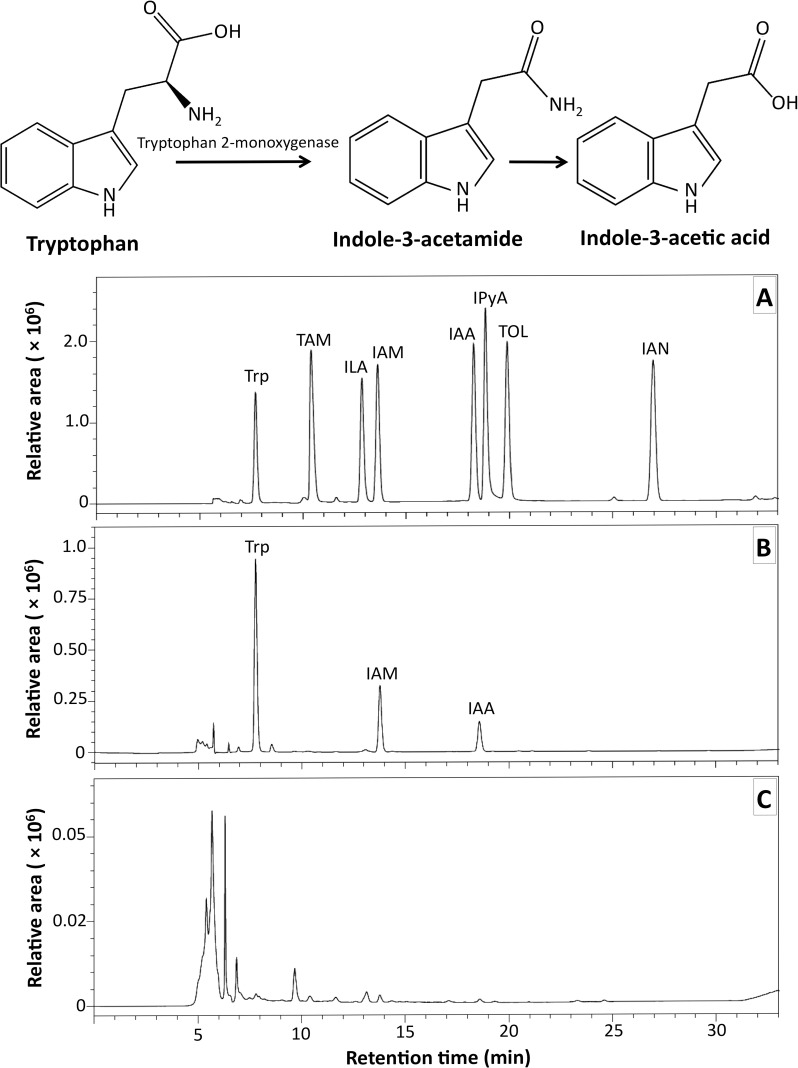
Tryptophan 2-monooxygenase activity of *Colletotrichum fructicola* CMU-A109 and high performance liquid chromatography detection. A. Indole compound standards, B. Reaction mixture of L-Trp and enzyme extract, C. Enzyme extract.

HPLC analysis of the enzyme reaction mixture indicated the formation of IAM and IAA at retention times of 13.9 and 18.7 min, respectively. This corresponds to the IAA and IAA standards (Fig 4), which indicates that the crude lysate of *C*. *fructicola* CMU-A109 has tryptophan 2-monooxygenase activity.

### Optimal conditions for fungal IAA production by *Colletotrichum fructicola* CMU-A109

**Fig 5:**
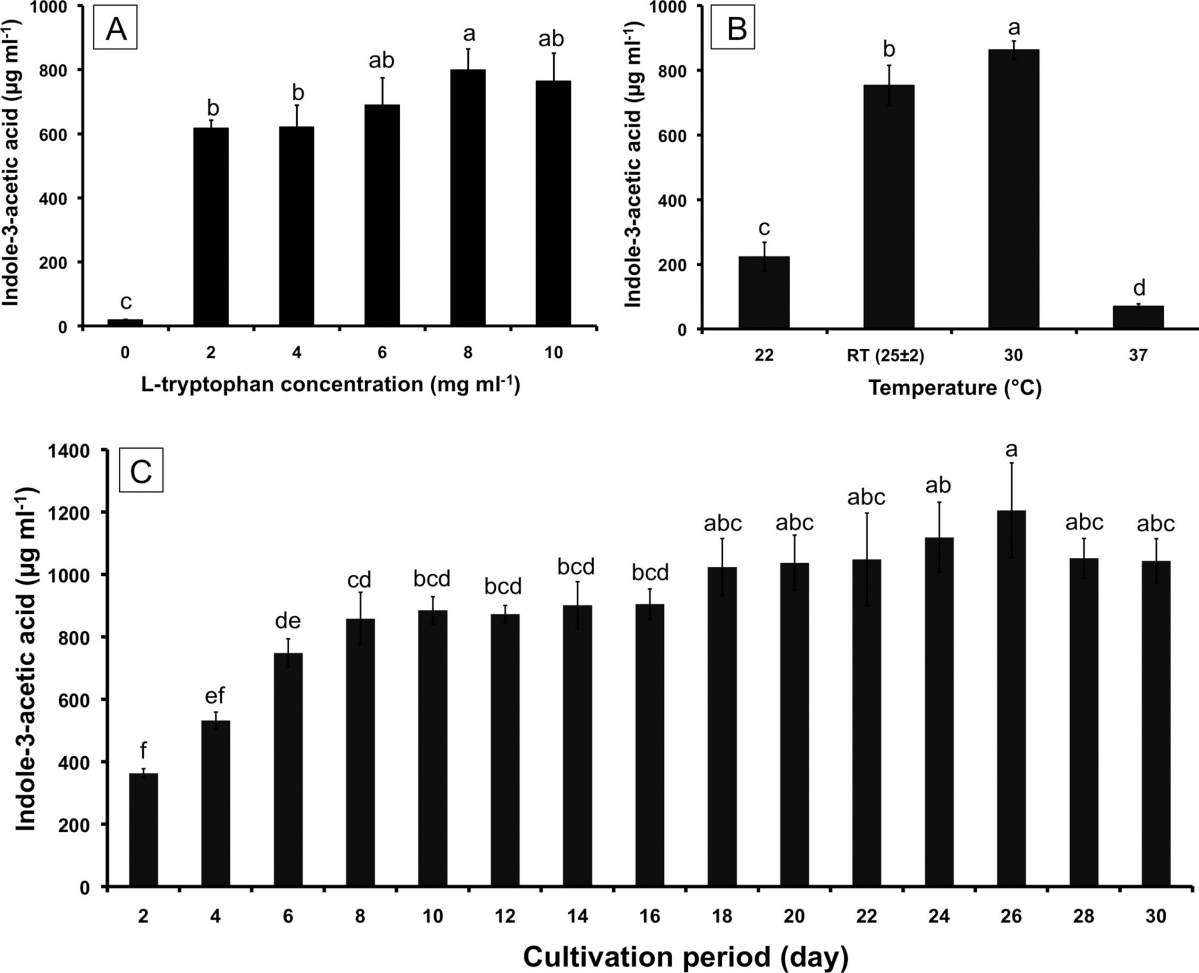
Effects of L-Trp concentration (a), temperature (b) and cultivation period (c) on fungal IAA production by *Colletotrichum fructicola* CMU-A109. The results are means of three replicates ± SD. Different letters above each bar in the same parameter indicate the significant difference (*P*<0.05).

Fungal IAA production was estimated with different concentrations of L-Trp by *C*. *fructicola* CMU-A109. The amount of fungal IAA is shown in Fig 5A. Maximum IAA production (799.18±65.00 μg/mL) was observed in the liquid medium supplemented with 8 mg/mL L-Trp when shaken in the darkness at room temperature for 7 days.

The IAA production by *C*. *fructicola* CMU-A109 at different temperatures is shown in Fig 5B. The results indicated that this fungus could produce IAA at all of the tested temperatures in this study and the maximum IAA yield (862.26±28.03 μg/mL) was observed at 30ºC, followed by that at room temperature (752.75±62.30 μg/mL). The lowest IAA yield (70.39±7.23 μg/mL) was recorded at 37ºC.

The effects of the cultivation period on the IAA production of *C*. *fructicola* CMU-A109 are shown in Fig 5C. The results indicated that the IAA yield increased from 0 to 26 days. The highest IAA yield was found (1205.58±151.89 μg/mL) at 26 days after cultivation.

### Biological activity of fungal IAA

**Fig 6:**
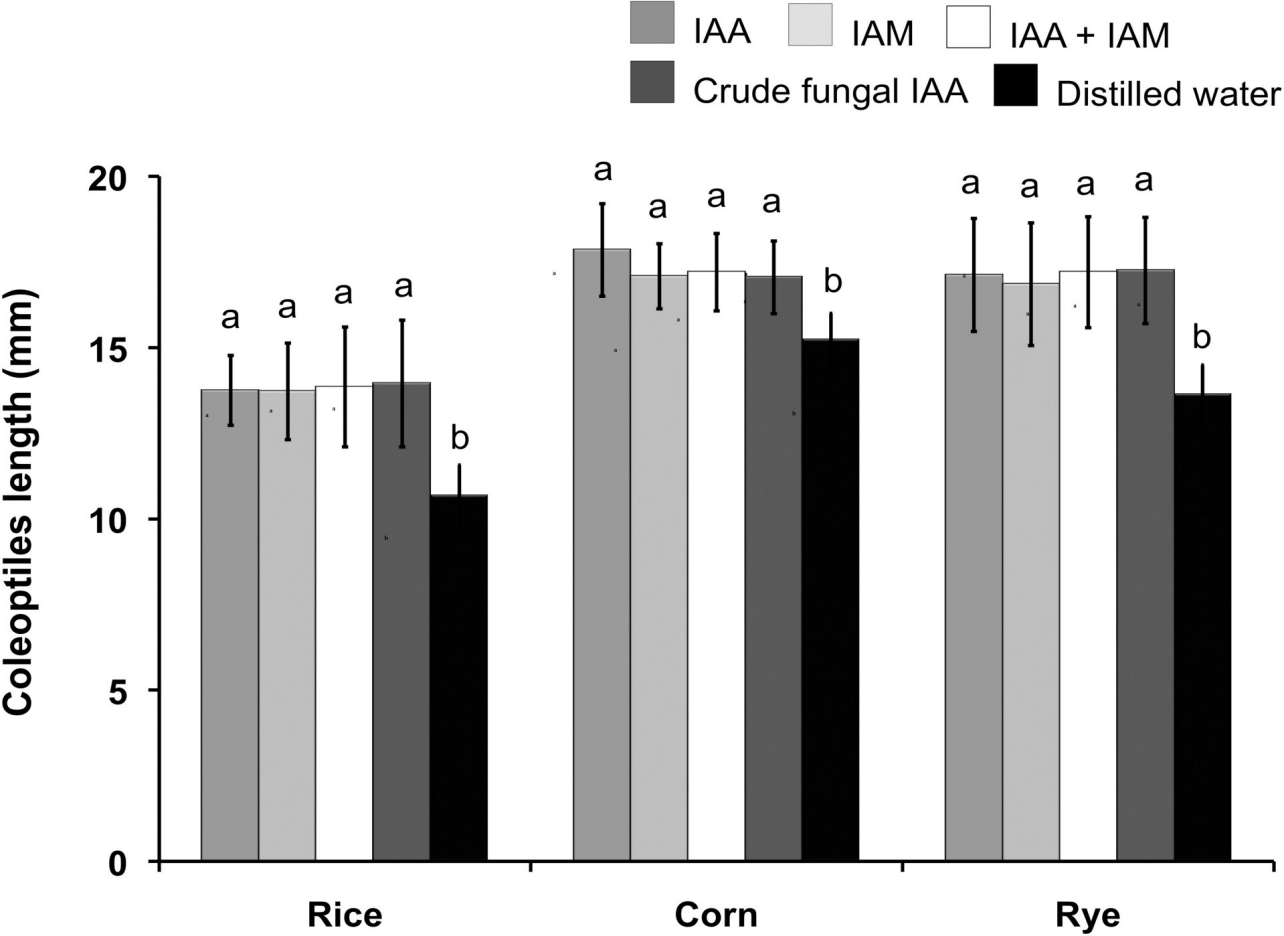
Coleoptile elongation of rice, corn and rye. Data are mean of five replicates. Error bar at each point indicates ± SD. Different letters above each bar indicates the significant difference (*P*<0.05). IAA = 20 μg/mL IAA treatment, IAM = 30 μg/mL IAM treatment, IAA+IAM = mixed 20 μg/mL IAA and 30 μg/mL IAM, Fungal IAA = crude fungal IAA treatment (contained 20 μg/mL IAA and 30 μg/mL IAM) and distilled water = distilled water treatment.

Elongation of rice, corn and rye coleoptile segments induced by crude IAA extract of *C*. *fructicola* CMU-A109 is shown in Fig 6. The lengths of all coleoptiles treated with fungal IAA were not statistically different from those of the IAA, IAM and IAA mixed IAM treatments. However, the values were significantly higher than the distilled water treatment.

## Discussion

IAA production by plant-associated microorganisms have been broadly studied and reported [7, 12, 20, 33]. In this study, *C*. *fructicola* CMU-A109 isolated from arabica coffee produced IAA in liquid medium supplemented with L-Trp at the IAA level 662.96 μg/mL. The results are similar to those of previous studies that found that the pure culture of endophytic fungi (e.g. *Alternaria alternata*, *Aspergillus fumigatus*, *Chaetomium globosum*, *Chrysosporium pseudomerdarium*, *C*. *acutatum*, *C*. *gloeosporioides*, *Fusarium* spp., *M*. *cinnamomi*, *Paecilomyces* spp., *Penicillium* spp., *Phoma* spp. and *Tulasnella* sp.) could produce IAA after being cultured in liquid medium supplemented with L-Trp [7, 10–13, 16, 17, 19, 49]. However, the present study provides the first report of IAA produced by *C*. *fructicola*. Indole derivatives as intermediate compounds give clues for identification of the IAA biosynthetic pathway. Reports on the IAA biosynthetic pathway of microbes are limited [22] and there is a need to more clearly understand the IAA production pathway of microorganisms. Consequently, a different biosynthesis pathway of IAA has been proposed for various microbial species [21, 22, 50] and it has been determined that a single species contained more than one pathway [22, 51]. Our study found that *C*. *fructicola* produced IAA from IAM, which was confirmed by the fungal IAA production from IAM and tryptophan 2-monooxygenease activities. Similarly, previous studies have reported synthesis of IAA via the IAM pathway in *C*. *gloeosporioides* f. sp. *aeschynomene*, *F*. *proliferatum*, *F*. *verticillioides*, *F*. *fujikuroi* and *F*. *oxysporum* [25, 31, 52]. However, Chung et al. [16] and Shilts et al. [53] reported synthesis of IAA by multiple pathways in *C*. *acutatum* (IAM and IPyA pathways). IAA production through the IPyA pathway was found in *Piriformospora indica*, *Rhodosporidium paludigenum*, *Rhizoctonia cerealis*, *R*. *solani*, *Ustilago maydis* and *U*. *esculenta* [26, 27, 30, 33, 54].

L-tryptophan concentration, temperature and cultivation period affected microbial IAA production. IAA produced by *C*. *fructicola* CMU-A109 increased when L-Trp concentration was increased to 8 μg/mL, IAA levels decreased when high concentration was used. This result is similar to those of previous reports, which indicated that the L-Trp concentration significantly affects microbial IAA synthesis. For example, Chutima and Lumyong [17] reported that the IAA level of *C*. *fructicola* CMU-AU 006 and *Tulasnella* sp. CMU-SLP 007 increased along with increased L-Trp concentrations but decreased after the L-Trp concentration reached 6 mg/mL. Bose et al. [55] found that the optimum L-Trp concentration for IAA produced by *Pleurotus ostreatus* was 1 mg/mL. Kumla et al. [20] reported that the maximum IAA level of different ectomycorrhizal fungi was found at levels of 2 mg/mL (*Astraeus odoratus*, *Pisolithus albus*, and *Scleroderma sinnamariense*) and 4 mg/mL (*Phlebopus portentosus*), which were optimal L-Trp concentrations. However, the concentration values decreased after adding L-Trp to the culture medium in higher amounts than the optimal L-Trp concentration. In this study, the optimal temperature for IAA production by *C*. *fructicola* CMU-A109 was 30ºC. This is in accordance with findings of previous studies, which reported that endophytic fungi normally produced IAA at temperatures between 25 to 30ºC [7, 11–13, 17, 50]. Moreover, 30ºC was the optimal temperature for IAA synthesis of a wide range of microbes such as *Lentinus sajor-caju* [56], *Streptomyces* sp. CMU-H009 [57], *Pantoea agglomerans* strain PVM [58] and some ectomycorrhizal fungi; *A*. *odoratus*, *Ph*. *portentosus*, *Pi*. *albus* and *S*. *sinnamariense* [20]. Our results indicated that the cultivation period affected the IAA production of *C*. *fructicola* CMU-A109. This result is supported by previous studies that have indicated that IAA production varies greatly among different microbial species, and our result also correlates with the finding that L-Trp availability in the culture medium is associated with microbial growth [20, 55, 57]. Moreover, the highest level of IAA was recorded when microbes were grown in the stationary phase [12, 20, 43]. Under the optimal conditions for *in vitro* IAA production, bacteria producing IAA ranged from 26.63 to 6100 μg/mL, respectively [57–61]. Previous reports have shown that the *in vitro* IAA level produced by fungi under the optimal conditions ranged from 40.76 to 563.80 μg/mL [12, 20, 32, 33, 55, 56, 62]. Interestingly, *C*. *fructicola* CMU-A109 produced IAA 1205.58±151.89 μg/mL under optimal conditions and its ability to produce IAA was highest when compared with previous reports on IAA produced by fungi.

Crude fungal IAA of *C*. *fructicola* CMU-A109 promoted the elongation of corn, rice and rye coleoptile segments. Similarly, IAA production by endophytic fungi (*C*. *gloeosporioides* CMU-AU 006, *Tulasnella* sp. CMU-SLP 007, *Tulasnella* sp. CMU-NUT 013 and *M*. *cinnamomi*) and ectomycorrhizal fungi (*A*. *odoratus*, *Pi*. *albus*, *Ph*. *portentosus* and *S*. *sinnamariense*) stimulated oat and rice coleoptile elongation [7, 12, 20]. In addition, the lengths of wheat coleoptile segments were significantly increased by IAA production from *Pleurotus ostreatus* [55].

## Conclusion

The endophytic fungus, *C*. *fructicola* CMU-A109 isolated from leaf tissues of coffee plant produced IAA *in vitro* via the IAM pathway. The highest IAA yield was obtained after 26 days of cultivation in liquid medium supplemented with 8 mg/mL L-Trp at 30°C. Crude fungal IAA stimulated coleoptile elongation as plays an important role in plant-growth promotion. Further study of this fungal isolate is required to evaluate its plant growth promoting abilities for the purposes of developing fungal inoculum. In addition, any pathogenicity needs to be tested.

## Supporting information

S1 Fig Identification of IAA produced by *Colletotrichum fructicola* CMU-A109 using intermediate indole compounds. A. Indole compounds standard, B. Cultivation with IAM, C. Cultivation with TAM, D. Cultivation with IPyA, E. Cultivation with IAN.
(TIF)
