# Supplementary figures and images for: Biosynthetic pathway and optimal conditions for the production of indole-3-acetic acid by an endophytic fungus, Colletotrichum fructicola CMU-A109
Source: PLoS One. 2018 Oct 18;13(10):e0205070. doi: 10.1371/journal.pone.0205070 (PMC6193638; doi:10.1371/journal.pone.0205070)

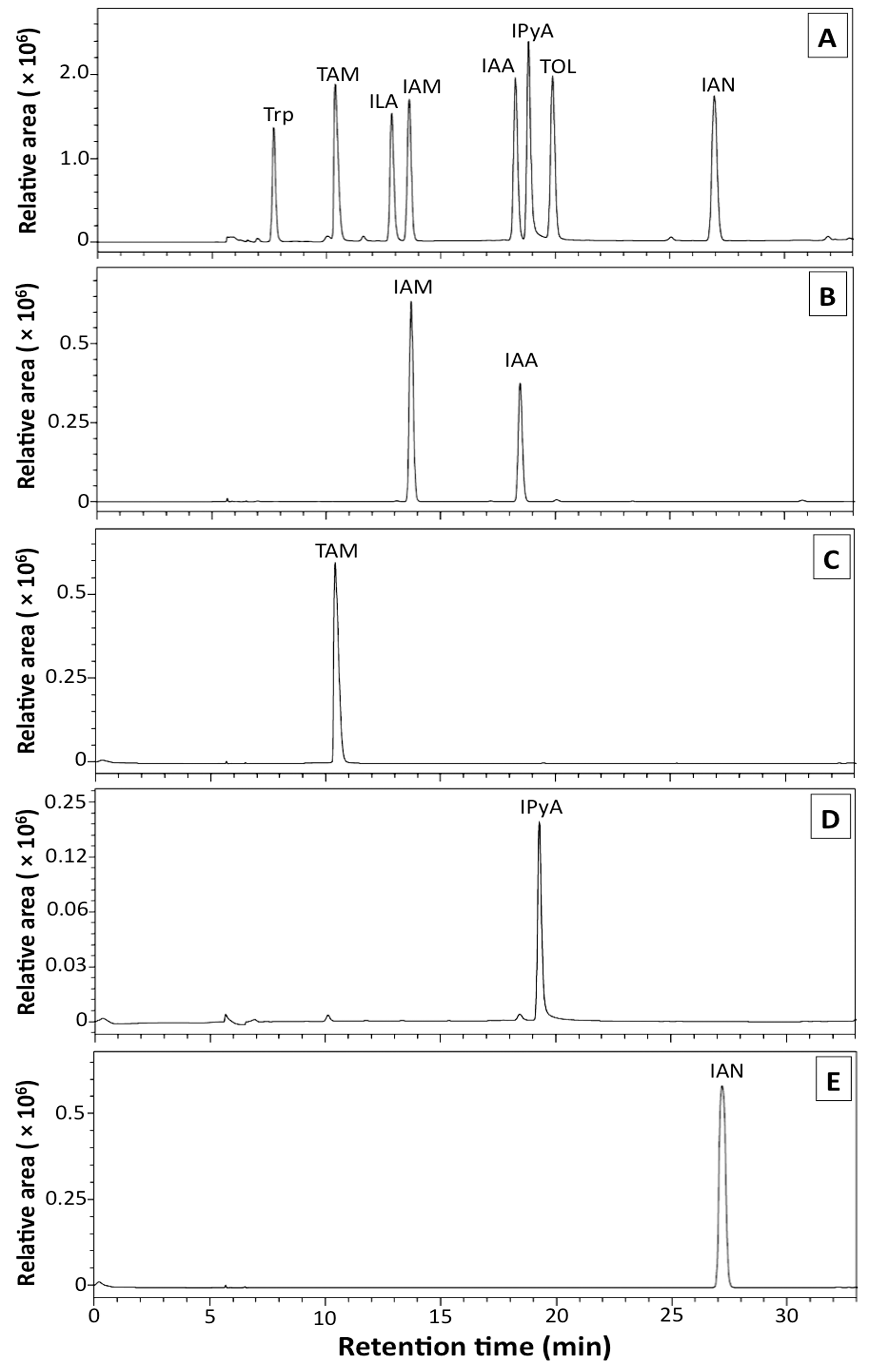

Supplement: S1 Fig — A. Indole compounds standard, B. Cultivation with IAM, C. Cultivation with TAM, D. Cultivation with IPyA, E. Cultivation with IAN. (TIF) [file pone.0205070.s001.tif]
